# Silk Fibroin Gels Fabricated from Ajisawa Reagent with Mechanochromic Sensing Capability

**DOI:** 10.3390/polym18151836

**Published:** 2026-07-27

**Authors:** Danila Maltsev, Cesare Proietti, Rocco Malaspina, Valeria Libera, Giorgio Schirò, Laura Ierimonti, Luca Valentini

**Affiliations:** 1 Civil and Environmental Engineering Department, University of Perugia, Strada di Pentima 4, 05100 Terni, Italy; danila.maltsev@dottorandi.unipg.it; 2 Department of Economics, Engineering, Society and Business Organization, University of Tuscia, Largo dell’Università s/n, 01100 Viterbo, Italy; cesare.proietti@unitus.it (C.P.); laura.ierimonti@unitus.it (L.I.); 3 Department of Physics and Geology, University of Perugia, Via A. Pascoli snc, 06123 Perugia, Italy; rocco.malaspina@dottorandi.unipg.it (R.M.); valeria.libera@unipg.it (V.L.); 4 CNRS, Institut de Biologie Structurale, 71 Avenue des Martyrs, 38044 Grenoble, France; giorgio.schiro@ibs.fr

**Keywords:** silk fibroin, secondary structure, gel, mechanochromic

## Abstract

Silk fibroin (SF) derived from *Bombyx mori* cocoons was employed to fabricate gels using Ajisawa reagent (AJ) as the extraction medium. The resulting regenerated SF solution was subsequently directly dialyzed against an ethanol–water mixture, inducing a gradual and controllable solution–gel transition. Obtained SF gels exhibit a pronounced increase in compressive strength aligned with rising initial fibroin concentration, along with excellent water stability. FESEM, FTIR, and synchrotron X-ray diffraction analyses reveal a homogeneous network structure, likely governed by uniformly distributed physical silk II crosslinks. SF gels containing Ca^2+^ ions predominantly retain the silk I conformation, consistent with optical transparency and higher ionic conductivity. Mechanochromic SF-based gel was further developed through a one-step process involving pentacosadiynoic acid (PCDA) infiltration, followed by UV-induced polymerization into polydiacetylene (PDA), yielding a blue-colored gel. Upon compression, the PDA-infused gel exhibits a clear color transition from blue to red, demonstrating avid mechanochromic behavior. This system represents a promising platform for Structural Health Monitoring (SHM), owing to its intrinsic self-reporting capability and direct visual detectability, enabling the effective sensing of stress-induced damage and crack formation.

## 1. Introduction

Nowadays, natural materials attract vigorous interest in biomedical and tissue engineering applications [1,2]. Among them, silk protein, extracted from silk cocoons biosynthesized by the *Bombyx mori* silkworm, has many useful properties, such as a low inflammatory response, controllable biodegradability, biocompatibility, and excellent mechanical properties [3,4,5,6,7,8]. Additionally, regenerated silk fibroin (SF) enables creation of functional materials [9,10,11] suitable for optical [12,13,14] and sensing applications [15,16,17,18].

Silk fibroin can be processed into a variety of physical forms, including films, gels, sponges, and micro-/nanoparticles. Among these, hydrogels are particularly attractive due to their mechanical and structural similarities to soft tissues and extracellular matrices [19,20]. However, most silk-based gels tend to dry rapidly under ambient conditions, leading to progressive hardening. This behavior is mainly associated with the use of volatile solvents, which gradually evaporate from the gel network. The consequent loss of solvent significantly alters the gel properties.

Recent studies have addressed this limitation by developing air-stable and conductive gels through the incorporation of glycerol or ionic liquids (ILs) within polymer crosslinked frameworks [21,22,23,24,25]. Nevertheless, these gels typically rely on covalently crosslinked networks, the fabrication of which involves complex and multi-step polymerization processes.

In contrast, physically crosslinked silk fibroin hydrogels can be readily obtained from aqueous silk fibroin (SF) solutions by inducing a conformational transition of the protein chains from random coil to β-sheet structures [26,27,28]. Despite this advantage, such physically crosslinked gels often exhibit suboptimal mechanical properties. This limitation arises from the intrinsic amphiphilic nature of silk fibroin chains, which promotes spontaneous aggregation in regenerated silk fibroin solutions. Therefore, the construction of a homogeneous network with uniformly distributed crosslinking domains (β-sheet crystallites) represents a promising strategy to enhance the mechanical performance of SF-based hydrogels.

The transition to β-sheet structure can be triggered by several stimuli, including shear forces, elevated temperatures, and water annealing [29,30]. Among chemical approaches, the use of organic solvents—particularly alcohols—has been widely explored due to their simplicity, availability, and low cost. For instance, Ishida et al. demonstrated that immersion of silk fibroin films in ethanol for 30 min induces a conformational transition from silk I (random coil) to silk II (β-sheet structure) [31]. This effect has been attributed to the dehydration action of alcohol molecules, which remove water surrounding the silk fibroin chains, thereby promoting intermolecular β-sheet formation and stabilization [32,33].

Although alcohol-induced gelation of silk fibroin has been previously reported, further investigation is needed to better control the resulting network structure and properties. In this context, the present study explores the use of Ajisawa reagent—composed of CaCl_2_, ethanol, and water in a 1:2:8 molar ratio—as an alternative starting point for the formation of SF-based gels.

In this system, water acts as a swelling agent, expanding the amorphous regions of silk fibroin, while ethanol penetrates the crystalline domains, reducing surface tension and weakening hydrophobic interactions. Calcium ions, in turn, exert a kosmotropic effect by blocking formation of interchain hydrogen bonds in SF hydrophobic regions, maintaining silk I conformation [34]. This synergistic effect leads to a reduction in β-sheet content, which plays key role in promoting silk fibroin gelation, should this balance be disrupted. This mechanism suggests that removal of Ca^2+^ ions from the dialysis cell serves as an effective strategy for increasing β-sheet formation, enhancing the performance and stability of silk gels without the need for silk modification.

To obtain SF gel with desired properties, the choice of the processing method and reagents is critical. Here, we describe the fabrication protocol of SF-based gels prepared directly from Ajisawa reagent solution of silk. This provides an opportunity to create a physically crosslinked SF by inducing gelation in Ajisawa solution against an ethanol/water solution. The presented procedure leads to gel formation right after the dissolution process of SF in Ajisawa ternary solvent, which obviates the necessity to dialyze against water first to obtain SF aqueous solution. For comparison, typical SF gelation protocols require at least 3 days for water dialysis and protein purification, and after that, another step for gelation of the aqueous solution [35]. The typical gelation time of SF aqueous solution is several weeks, depending on various parameters such as concentration and temperature [36]. This gelation time can be reduced to several hours by adding alcohol or a surfactant [37,38]. In this work, we demonstrate the ability to derive, without any sort of chemical addition, a fully crosslinked silk gel within 48 h from the dissolution process, thus reducing the processing time of existing protocols by at least a day.

Based on the investigation and understanding of the structure and properties of SF-based materials, we have developed a gel that combines promising mechanical properties and water stability. To take a step further in the direction of possible practical application, we suggest that with simple infiltration of pentacosadiynoic acid (PCDA) and its subsequent UV-conversion to a polydiacetylene (PDA) network, such gels may be endowed with a visible colorimetric transition in response to compressive forces. We attribute the markedly higher activation threshold of PDA–SF gels to elastic buffering and mechanical confinement imposed by the stiff, β-sheet-rich silk matrix, which transmits stress to the mechanophore PDA network encapsulated architecture.

## 2. Experimental Section

### 2.1. Materials

*Bombyx mori* silkworm cocoons were purchased from Amazon. Absolute ethanol, anhydrous calcium chloride, sodium bicarbonate, and 10,12-pentacosadiynoic acid were obtained from Merck KGaA (Darmstadt, Germany). All chemicals were of analytical grade and used as received without further purification.

### 2.2. Preparation of Regenerated Silk Fibroin in Ajisawa Reagent

Silk cocoons were cut into strips of approximately 5 mm in width and degummed by boiling in a 0.3 M NaHCO_3_ solution for 30 min. The fibers were subsequently rinsed and soaked in deionized water three times for 15 min each, to ensure complete removal of sericin. After air-drying for 48 h, the degummed fibers were cut and weighed according to the desired final concentrations (50, 100, and 150 mg/mL) and placed into glass vials. Ajisawa reagent (AJ) was prepared beforehand by mixing water, absolute ethanol, and calcium chloride in a molar ratio of 8:2:1. The salt was added gradually under vigorous stirring to prevent excessive heating of the mixture. Once combined with AJ, the vial was left at room temperature for 20 min and then heated at 80 °C for 3 h, resulting in a transparent and viscous solution. For all formulations, residual aggregates were dispersed by magnetic stirring, yielding a homogeneous suspension.

### 2.3. Preparation of SF-Based Gels

**Figure 1:**
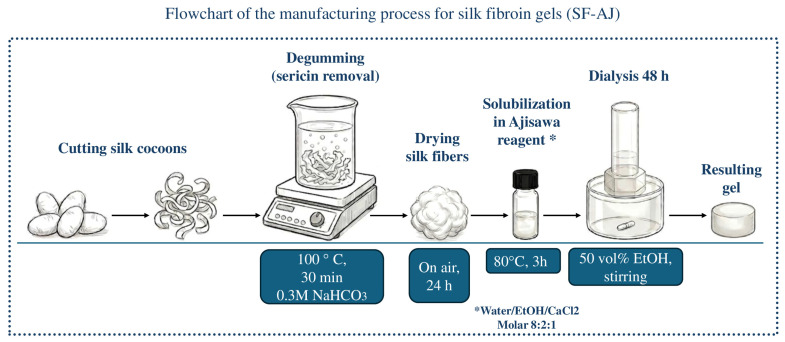
Schematic diagram of the procedure used to prepare silk fibroin gels (SF AJ). The silk cocoons are cut and degummed in a NaHCO_3_ solution (0.3 M, 100 °C, 30 min) to remove the sericin. The resulting fibers are air-dried for 24 h and subsequently solubilized in Ajisawa reagent (H_2_O/EtOH/CaCl_2_, molar ratio 8:2:1) at 80 °C for 3 h. The solution is then dialyzed for 48 h against a 50% aqueous ethanol solution under agitation, yielding the final gel.

SF AJ solutions were transferred into mini dialysis units (Cytiva, Wilmington, DE, USA) and dialyzed against a 50 vol% ethanol/water mixture for 48 h at room temperature (20 °C) under continuous stirring (Figure 1).

At the end of dialysis, the gels were removed from the tubes, measured, and characterized. Gels stored in 50 vol% ethanol to prevent dehydration prior to analysis are hereafter referred to as *fresh*. Before testing, they were removed from the solution, gently blotted with paper tissue, and allowed to air-dry for a few minutes.

Samples left to dry in ambient conditions are referred to as *dried* gels. In this case, complete drying required 72 h in air to reach constant mass and dimensions. From this point onward, both fresh and dried gels are denoted referencing their initial SF concentration, i.e., SF 50, SF 100, and SF 150 correspond to gels derived from 50, 100, and 150 mg/mL SF solutions.

While gelling occurs as a front starting near the membrane and traveling outwardly through the contents of the tube, there occurs a volume of transparent gel furthest away from the membrane. While the physical gelation is caused by lowering concentration of Ca^2+^ ions as they diffuse towards the dialysis bath following the gradient, the mentioned clear phase can be shielded from the diffusion gradient pull by the network of compact gel between itself and the membrane—thus retaining higher concentration of those ions (Figure S1 in the Supporting Information).

### 2.4. Water Swelling Tests

In water stability tests, fresh and dried SF 50, 100, and 150 gels were submerged in deionized water at room temperature and removed at predetermined exposure times to be immediately weighed and measured. This procedure was repeated in triplicate to ensure reliable results. Two different ratios were obtained from these gravimetric measurements,

QF = (m_S_ − m_F0_)/m_F0_ (1)

and

QD = (m_S_ − m_D0_)/m_D0_, (2)

where m_S_ is the weight of a swollen gel, and m_F0_ and m_D0_ correspond to the weights of the fresh and dried gels, respectively.

### 2.5. Mechanical Characterization

The mechanical properties of SF-based gels were evaluated using an Instron 5565 universal testing machine (Instron, Norwood, MA, USA) equipped with a 50 kN load cell with 1 N sensitivity. Uniaxial compression tests were carried out at room temperature. Cylindrical specimens (diameter 14 mm, height 10 mm) were tested at the compression rate of 1 mm min^−1^, until set displacement of 5 mm (50% compression). Stress–strain curves were obtained, and compressive and Young’s moduli determined from the linear region of the stress–strain curves (0–20%, or 20–40% strain). At least three independent specimens were tested for each formulation.

### 2.6. Fourier Transform Infrared Spectroscopy (FTIR)

ATR-FTIR spectra were acquired using a JASCO FT/IR-4X spectrometer equipped with a high-luminosity ceramic source, a Michelson interferometer with a Ge/KBr beamsplitter, and a DLATGS detector (JASCO Corporation, Tokyo, Japan). Measurements were performed directly on dried gels over the spectral range of 4000–500 cm^−1^ with a resolution of 4 cm^−1^. Background spectra were recorded prior to sample analysis. Each spectrum represents an average of 50 scans.

Analyses of SF gels’ secondary structure were performed by fitting the Amide I signal of SF. The fitting procedure largely resembles the protocol published in the literature [39], which proved to deliver robust and consistent results. A straight baseline was defined under Amide I band (1720–1590 cm^−1^). A combination of 6 Gaussian profiles was utilized to fit the experimental data. The initial value of the peak position of each Gaussian was defined according to [39] and was let to vary within a range of 5 cm^−1^ below or above the set point. The width of each component was allowed to vary between 5 and 15 cm^−1^ in order to avoid highly overlapping bands. There was no restriction for the amplitude of each Gaussian, apart from the obvious condition that it should be nonnegative. Each Gaussian signal is assigned to particular secondary structure component. That secondary structure content is then derived as the ratio between the respective Gaussian area and the total reconstructed Amide I band area, multiplied by 100 for the percentage. The fitting procedure and all subsequent calculations were performed using a custom Python script (version 3.11.14, making use of the libraries NumPy version 2.3.6 and SciPy version 1.17.1).

### 2.7. Field-Emission Scanning Electron Microscopy (FESEM)

The morphology of the gels was examined using a LEO 1525 FESEM system (Carl Zeiss Microscopy GmbH, Jena, Germany) equipped with a ZEISS angle-selective backscattered electron detector. Measurements were performed at accelerating voltage of 5 kV.

### 2.8. Synchrotron Radiation X-Ray Diffraction

XRD measurements were carried out on the BM07 beamline at the European Synchrotron Radiation Facility (ESRF, Grenoble, France). Both fresh and dried gels were analyzed using an incident beam energy of 12.658 keV (corresponding to a wavelength of 0.980 Å) and a beam cross-section of 0.2 × 0.2 mm. The beamline is equipped with a DECTRIS Pilatus3 6 M detector, and the sample-to-detector distance was set to 390 mm. Samples were placed inside Mylar capillaries and mounted on the goniometer using a magnetic pin. The diffraction pattern of the empty capillary was recorded prior to sample measurements. Each dataset consists of 120 diffraction images collected through consecutive 3° rotations, covering a full 360° rotation. The 2D diffraction patterns were converted into 1D profiles by azimuthal integration using the open-source Python library pyFAI (version 2026.5.0). The 120 diffraction patterns associated with each sample were then averaged.

### 2.9. Synthesis and Characterization of PDA-Functionalized Gels

A mechanochromic SF-based gel was successfully developed by incorporating a chromogenic polydiacetylene (PDA) component through a one-step fabrication process. This approach involves the infiltration of pentacosadiynoic acid (PCDA) precursors into the SF gel, followed by UV-induced polymerization to generate a conjugated PDA network, resulting in a characteristic blue–purple coloration. To optimize PDA incorporation within the silk fibroin matrix, a systematic functionalization study was carried out by progressively investigating the influence of PCDA concentration, silk fibroin concentration, infiltration time, gel hydration state, solvent exchange, and solvent evaporation prior to UV irradiation. The tested protocols are summarized in Table 1.

## 3. Results and Discussion

### 3.1. Morphology and Mechanical Properties of SF-Based Gels

SF gels were prepared from *Bombyx mori* cocoons using Ajisawa reagent as the extraction medium, ensuring efficient dissolution while preserving the molecular integrity of SF. The regenerated SF solution was subsequently dialyzed against an ethanol–water mixture of fixed concentration—with equal starting volumes of 96% EtOH and water—which induced a controlled sol–gel transition through the formation of physical crosslinks (Figure 1).

**Figure 2:**
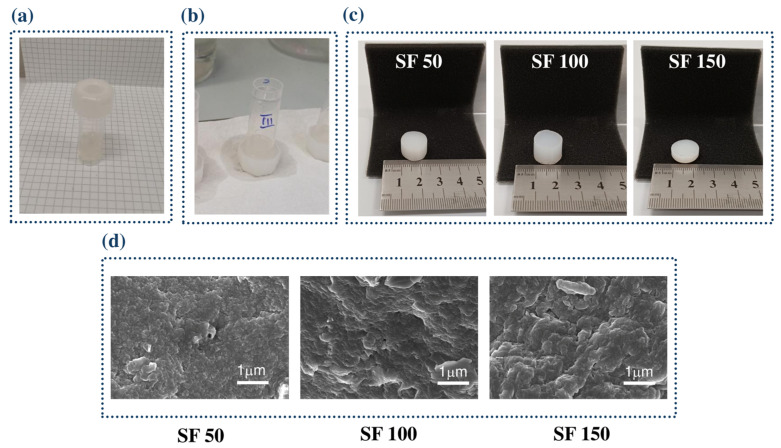
Visual and mechanical characterization of SF gels. Demonstration of the facile production of silk gel—shown as silk solution (**a**), silk gel in a dialysis tube (**b**), and extracted gel of various initial concentrations of silk (**c**). (**d**) FESEM images of the respective dried samples.

To report the physical properties of SF gels, their stability and mechanical properties were characterized (Figure 2). SF solutions were cast in cylindrical dialysis tubes (14 mm diameter) (Figure 2a). Following the dialysis, SF gels maintained their original dimensions (Figure 2b). This is likely due to the highly hygroscopic profile of the gels, which allows water retention.

The morphology of SF-based gels was investigated by FESEM (Figure 2d), revealing a highly homogeneous network architecture. Morphological analysis also highlighted a uniform and continuous matrix, without evident phase separation or aggregation phenomena. The SF 100 and SF 150 samples appear marginally more textured, but the fundamental structural consistency remains equal for all the gels.

**Figure 3:**
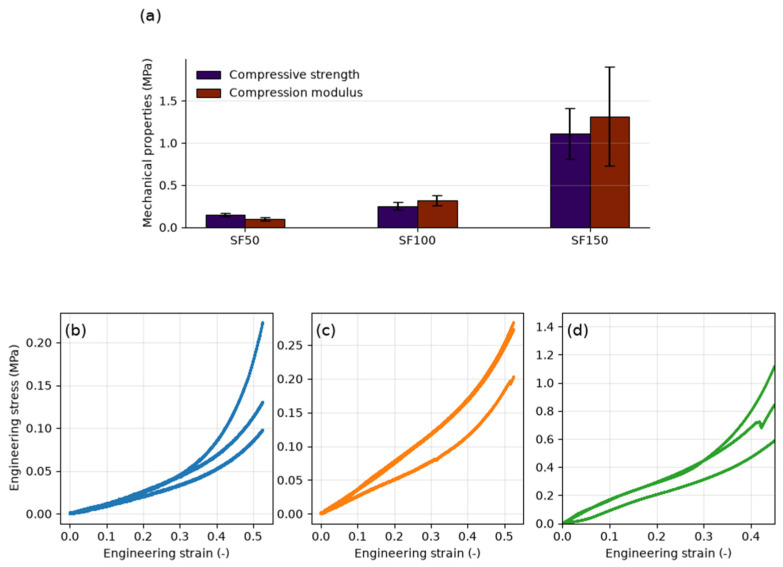
Mechanical characterization of SF gels prepared at different fibroin concentrations. (**a**) Compressive strength and modulus (mean ± standard deviation) of the SF 50, SF 100, and SF 150 samples. (**b**–**d**) Representative engineering stress–strain curves obtained during uniaxial compression tests for the SF 50 (blue), SF 100 (orange), and SF 150 (green) samples, respectively. An increase in silk fibroin concentration leads to a progressive increase in both the compressive strength and stiffness of the material, with the SF 150 sample exhibiting elevated compressive strength and compression modulus.

The SF 50 and SF 100 gels exhibited compressibility up to 60%, showing a compressive strength of 200 kPa (Figure 3) without breaking. However, the 150 SF gel demonstrated lower deformability (50%) with an increase in compressive modulus and strength.

### 3.2. Structure of SF-Based Gels

**Figure 4:**
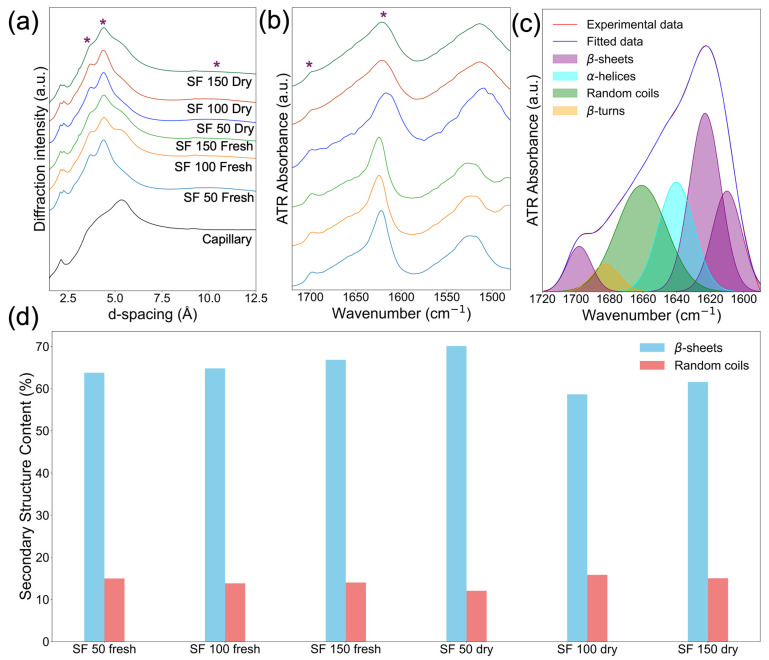
(**a**) XRD profiles of all the gels, with the capillary reference curve below (used as sample holder). Typical Silk II regions are denoted by asterisks. (**b**) FTIR spectra of the samples, retained in ATR configuration. The curves follow the same ordering and coloring as the previous panel. Asterisks (*) show the signals associated with the Silk II conformation in XRD and FTIR data. (**c**) Example of the deconvolution of the Amide I band with Gaussian components. (**d**) Results of the deconvolution analysis, showing the relative β-sheet vs. amorphous content.

The structural organization of SF-based gels was investigated by FTIR spectroscopy and synchrotron XRD (Figure 4). The comparison with spontaneously gelled reference aqueous SF solution is presented with a photograph of the gel and its FTIR spectrum.

At the molecular level, FTIR and XRD analyses confirm that β-sheet structures represent the dominant secondary conformation. Specifically, XRD profiles display the typical signatures of the Silk II structure, with characteristic Bragg reflections at 3.7, 4.4, and 9.8 Å (Figure 4a, evidenced by asterisks) [40]. The other peaks present in the diffraction profiles are associated with the capillaries used as sample holders during data acquisition, as they correspond to the reference profile of the empty capillary, which is reported for comparison.

These results are in good agreement with the previously reported characterization of silk II materials. For example, Sinsawat et al. [41] found similar values, from both experimental and theoretical standpoints, for the crystal structure of antiparallel β-pleated sheet nanocrystals constituting the silk fibers. They estimated a crystalline fraction of the silk fibers in the range of 60–65%, based on equatorial reflections, with the fiber perpendicular to the X-ray beam.

These findings are further corroborated by FTIR spectroscopy (Figure 4b). Both Amide I (1700–1590 cm^−1^) and Amide II (1570–1490 cm^−1^) regions are highly indicative of the protein secondary structure [42,43]. In particular, the bands at 1622 and 1700 cm^−1^ of the Amide I profile, marked by asterisks, clearly show the formation of antiparallel β-sheet domains. Deconvolution of the Amide I band (Figure 4c) for all gel samples reveals β-sheet content ranging from 60% to 70% (Figure 4d), values close to those reported for silk fibers, confirming the highly ordered nature of the network, where the β-sheet nanocrystals likely provide physical crosslinks that ultimately lead to SF gelation.

The formation of β-sheet structure in silk-based materials is a complex phenomenon which can be affected by the chemical environment, pH, and temperature, and can govern both the optical and mechanical properties of the resulting material. Hu et al. [44] studied the isothermal crystallization of SF by both infrared and X-ray synchrotron radiation. Their results prove that SF can be modeled as a block copolymer, with an interplay between hydrophilic and hydrophobic regions of the protein chain that eventually results in the formation of nanocrystals.

Moreover, it is reported in the literature that silk fibroin films obtained from a regeneration protocol in FA/CaCl_2_ solution can develop a mixed Silk I and II conformation after water exposure [45]. These findings support our work in highlighting the important role calcium ions play in controlling silk conformation. In this sense, our dialysis protocol provides a more controlled and reliable way to release calcium ions into the bath following the concentration gradient, thus permitting better removal of the ions. This fact, along with the presence of ethanol in the dialyzing solution at the same concentration as the original Ajisawa ternary solvent [38], warrants formation of the gel.

To further drive this claim home, the dialysis protocol was replicated using 3%, 6%, and 10% CaCl_2_ in the dialysis bath, while keeping the same composition of EtOH and water. This imposes control over the calcium ion gradient and gradually decreases it with respect to the original protocol while maintaining the same ethanol concentration, ensuring that the salt content is the only variable. Photographs, as well as FTIR curves and deconvolution percentages of the resulting gel samples, are provided in Figure S2, panels (a), (b), and (c), respectively. The results testify that for 3% and 6% salt concentration, the dominant secondary structure component remains silk II, but to a lower degree than for the gels produced in absence of CaCl_2_ in the dialysis bath. Moreover, 10% CaCl_2_ in the bath hinders the gelation even after several days, confirming the critical role of calcium ions in regulating the properties of the SF gels.

### 3.3. Water Stability of SF-Based Gels

The SF-based gels exhibit excellent stability in aqueous environments, primarily due to the presence of physically crosslinked crystalline domains, as evidenced in the previous section. These regions reduce the solubility of fibroin chains and prevent structural disintegration upon prolonged exposure to water. The ethanol-induced gelation process promotes the formation of stable intermolecular interactions, effectively limiting excessive swelling and dissolution. As a consequence, the gels retain both their structural integrity and mechanical performance under hydrated conditions.

This good water stability is particularly advantageous for applications in humid or fully aqueous environments, where conventional physically crosslinked hydrogels often suffer from rapid degradation or loss of mechanical properties. The balance between hydrophilicity and structural cohesion ensures both water compatibility and durability.

The SF 100 and SF 150 fresh gels (i.e., gels not subjected to drying) indicate good water stability, based on the results of 7-day-long immersion. Fluctuations in their mass in the first several days should largely be attributed to the substitution of the ethanol–water medium remaining enclosed by the end of dialysis, for water (which has a slightly higher density—1 g/cm^3^ vs. 0.94 g/cm^3^ for the dialysis bath ethanol–water solution). However, SF 50 fresh gel proved not quite as durable, losing 17% of the mass prior to stabilization. This behavior can be broken down into two concurrent processes: (i) diffusion-driven replacement of the post-dialysis ethanol–water mixture with pure water, and (ii) partial leaching of shorter polymeric chains [46]. For SF 50, the lower density of the protein and lower number of crosslinks, in conjunction with higher porosity, led to much stronger detrimental influence. This suggests that somewhere between 50 and 100 mg/mL concentration of silk in precursor SF AJ solutions, there is a threshold that allows securing a water-stable gel. This has been corroborated by controlling the porosity of the gels. Considering swelling isometric, and utilizing linear dimensions of the samples, for fresh SF 150 gel it amounted to 79%, while for SF 100 and SF it amounted to 50–95 and 96%, respectively.

Porosity was calculated using the following simple equation:

(3) $$\rho_{gel}\;=\;\varphi_{silk}\times \rho_{silk}\;+\;\varphi_{solvent}\times \rho_{solvent}$$

where *φ* is the volume fraction of the respective component. The silk density figure for high β-sheet content was adopted from Reizabal et al. [47].

In contrast to their counterparts, dried SF 100 and 150 samples exhibit a mass increase of 15–35% upon immersion, due to partial rehydration and expansion of the porous network. SF 50 follows the same path during the initial swelling, but then diverges and loses mass due to the network’s accessibility and its susceptibility to the release of shorter polymeric chains, as discussed above.

**Figure 5:**
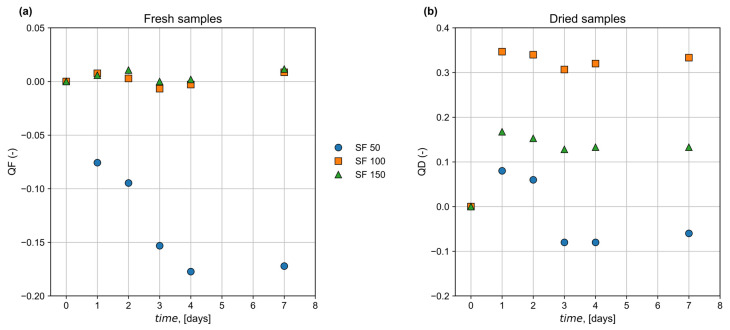
Mass change upon immersion in water under ambient conditions: (**a**) for fresh gels and (**b**) for dried ones.

For dried gels, where all the solvent has left the system (as confirmed by a measured lack of ionic conductivity), the porosities were as follows: 21% for SF 50, 15% for SF 100, and 18% for SF 150. However, during swelling, some of the original porosity of fresh gels was recovered—prominently so for SF 100. This may be explained by its balance of structural cohesion, not susceptible to degradation (as shown in Figure 5a), while offering larger initial porosity and easier water access.

### 3.4. PDA Functionalization and Mechanochromic Response of SF-Based Gels

#### 3.4.1. Preliminary Test

**Figure 6:**
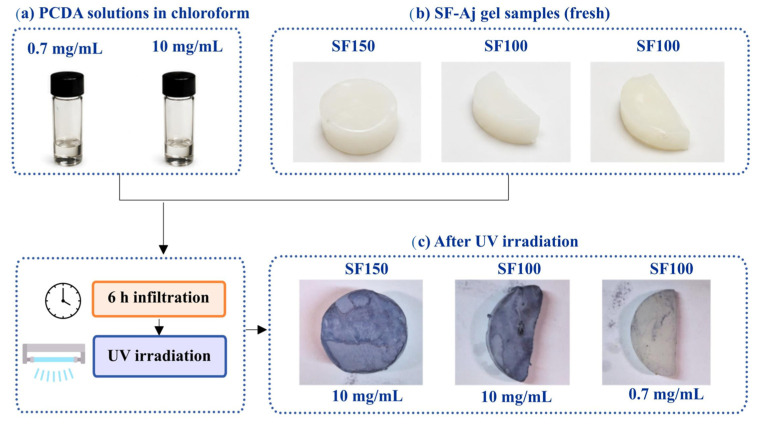
Preliminary test to verify the infiltration of PCDA-chloroform solution into SF gel. (**a**) Two concentrations of PCDA-chloroform (0.7 and 10 mg/mL); (**b**) fresh gels before infiltration; (**c**) gels after 6 h of infiltration and subsequent UV irradiation.

Preliminary experiments were performed on fresh SF gels to evaluate the influence of both pentacosadiynoic acid (PCDA) concentration and silk fibroin content on monomer infiltration. Two SF 100 specimens and one SF 150 specimen were immersed in PCDA-chloroform solution for 6 h. The two concentrations tested were 0.7 and 10 mg/mL. Following infiltration, the samples were irradiated with UV light to induce polymerization of PCDA into PDA (polydiacetylene). This protocol was labeled P1, as reported in Table 1 and Figure 6. Visual inspection of the resulting coloration was used as a qualitative indicator of successful infiltration and polymerization. Based on the stronger blue coloration observed for the 10 mg/mL PCDA solution and for the SF 150 specimens, respectively, the subsequent experiments were conducted using SF 150 gels and higher PCDA concentration.

That demonstrated that both PCDA concentration and silk fibroin content can influence the efficiency of PDA functionalization. While increasing the PCDA concentration expectedly leads to a denser PDA network and color response, the not-quite-so-intuitive stronger coloration of SF 150 suggests that a denser silk network can retain a larger amount of infiltrated monomer. These observations identified SF 150 combined with 10 mg/mL PCDA as the most promising formulation.

#### 3.4.2. Optimization of Infiltration Time

**Figure 7:**
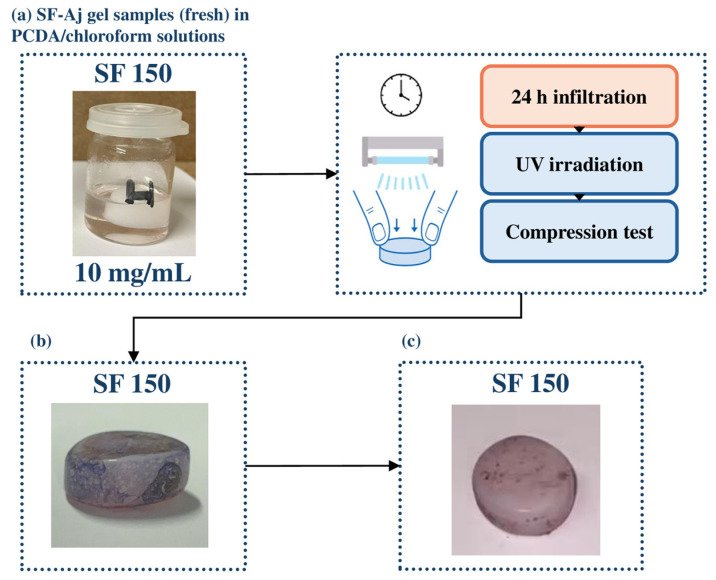
Effect of prolonged infiltration time on the functionalization of fresh SF-based gels (Protocol P2). (**a**) Fresh SF 150 gel immersed in a 10 mg/mL PCDA–chloroform solution and subjected to 24 h of infiltration, followed by UV irradiation and manual compression. (**b**) Blue staining of the gel after UV-induced polymerization of PCDA into PDA. (**c**) Appearance of the gel after compression, revealing only a superficial red color transition, indicating that prolonging the infiltration time alone does not guarantee effective penetration of the PCDA solution.

Based on the results obtained in Protocol 1, a second series of tests was carried out to assess the effect of a longer infiltration time on the absorption of PCDA within the silk fibroin matrix (Figure 7). Given the more intense coloration observed for SF 150 samples and in the solutions with higher monomer concentration, SF 150 gels and 10 mg/mL PCDA-chloroform solution were selected for the subsequent tests.

A fresh SF 150 sample was immersed in PCDA solution for 24 h. After infiltration, the sample was taken out and left to air dry for 5 min to allow partial evaporation of residual chloroform. The gel was then irradiated with UV light to induce PCDA polymerization and the formation of corresponding PDA.

Following UV irradiation, the sample developed a visible blue color, suggesting the presence of PDA on the surface. However, during visual inspection, a significant portion of the PDA was simply smeared by lab gloves, leaving the sample blank in the contact area.

Examination of the sample after compression revealed only a thin superficial region with a reddish tinge, indicating that infiltration had occurred mainly in the outermost layers of the gel. These results suggest that increasing the immersion time from 6 h to 24 h is not sufficient to ensure effective penetration of PCDA-chloroform into fresh gels.

One possible explanation relates to the high water content in the fresh samples. Since PCDA is carried by chloroform, a solvent that is poorly miscible with water, the presence of an aqueous phase within the gel could significantly limit the diffusion of the monomer, facilitating surface deposition instead. Based on that, the subsequent experiments were conducted using pre-dried samples.

#### 3.4.3. Investigation of the Effect of Water

**Figure 8:**
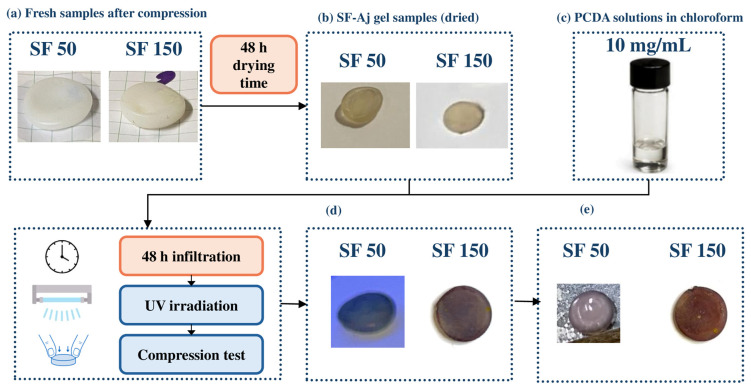
Investigation of the effect of water removal on the functionalization of silk fibroin gels with PDA (Protocol P3). (**a**) Fresh SF 50 and SF 150 samples after compression testing with an Instron machine. (**b**) The same samples after 48 h of drying under ambient conditions. (**c**) PCDA solution in chloroform (10 mg/mL) used for infiltration. (**d**) Appearance of the samples after UV irradiation, with PDA formation. The SF 150 sample exhibits a more intense blue color than SF 50, indicating greater PCDA infiltration and more effective PDA formation. (**e**) Appearance of the samples after compression. The SF 150 sample shows a more pronounced color transition from blue to red than SF 50, indicating a more pronounced mechanochromic response.

To investigate the influence of the gel’s residual water on PCDA infiltration, previously dried SF gels were employed. Dried SF 100 and SF 150 were selected from the samples previously used for mechanical testing (Section 3.1) and allowed to dry under ambient conditions for 48 h until dimension stabilization. Dried specimens were subsequently immersed in 10 mg/mL solution of PCDA in chloroform for 48 h to promote monomer infiltration. After immersion, the samples were removed from the solution and exposed to air for 5 min to allow partial evaporation of the solvent. UV irradiation was then applied for 15 min on each side to induce the polymerization of PCDA into PDA. The steps of protocol P3 are summarized in Figure 8. Following UV exposure, the dried gels exhibited a markedly more intense blue coloration compared to fresh gels subjected to the same treatment conditions, suggesting enhanced PCDA uptake. The functionalized samples then underwent qualitative compression tests by applying manual compression between two steel blocks. Visible color transition was observed after loading, providing preliminary evidence of a mechanically induced chromatic response in the PDA-functionalized silk fibroin gels. This behavior suggests that better removal of water facilitates the penetration of the chloroform-based PCDA solution into the porous silk network, allowing more efficient formation of the PDA phase throughout the material.

#### 3.4.4. Effect of Drying and Prolonged Solvent Evaporation on the Mechanochromic Response

Following the results obtained in P3, further tests were carried out to assess the influence of longer chloroform evaporation time on the behavior of PDA-functionalized gels. To this end, two samples of SF 100 and SF 150 were left to air-dry for 48 h. Subsequently, the samples were immersed in 10 mg/mL PCDA-chloroform solution for 48 h to facilitate the infiltration of the monomer into the polymer matrix. Once the infiltration was complete, the samples were left to air-dry for 24 h. The gels were then UV-irradiated to yield PDA.

**Figure 9:**
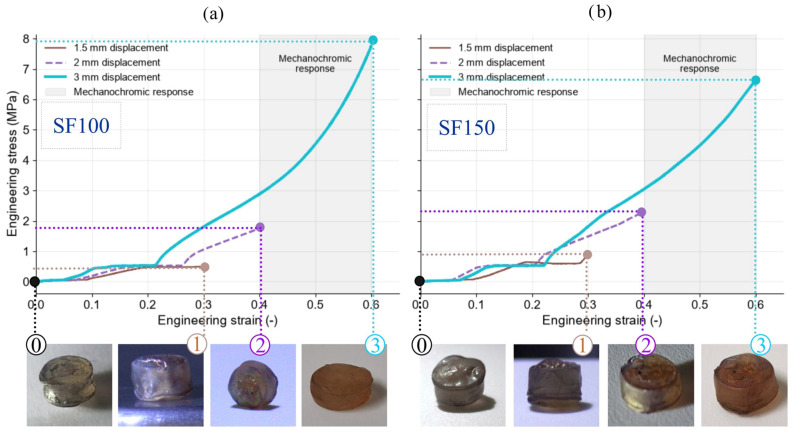
Engineering stress–strain curves obtained from sequential compression tests carried out on silk fibroin gels functionalized with PDA: (**a**) SF 100 and (**b**) SF 150. For each sample, three maximum displacement levels (1.5, 2 and 3 mm) were applied. Below the respective curves are images of the samples before compression (0) and after each compression step (1–3). The tests were carried out for qualitative purposes, with a view to identify the strain range in which the color change appears. An appreciable color shift was typically noted at 2 mm displacement. At the 3 mm level, all samples indicated a clear shift, with the SF 100 response more uniform than that of SF 150.

Both samples developed the characteristic blue color associated with PDA formation, indicating that the monomer successfully infiltrated and got polymerized. To evaluate the material’s mechanochromic response, the samples were tested using an Instron compression cell. The initial heights of the SF 100 and SF 150 cylindrical specimens were 4.8 and 6.1 mm, respectively. Due to this, the compression test protocol is described in terms of imposed displacement (1.5, 2 and 3 mm), corresponding to the actual load levels applied during the tests. For each sample, controlled displacements of 1.5, 2 and 3 mm were adopted, to correlate the extent of deformation applied and recorded load with the possible onset of color transition (Figure 9).

Both formulations exhibited a marked mechanochromic response; however, the SF 100 sample showed a more uniform and almost complete transition compared to the SF 150 sample, which retained some blue areas after the third compression step. Given the qualitative nature of these tests, the differences in engineering stress and strain must be interpreted with caution. Natural geometric variability of the samples, surface irregularities and their positioning between the compression plates may influence the calculated mechanical parameters. Accordingly, the imposed displacement was deemed the most appropriate parameter to describe the successive loading steps. The main outcome of these tests is not a quantitative comparison of the mechanical properties of the two formulations, but rather the qualitative identification of the strain range reliably causing mechanochromic response. A more quantitative proof of the change in color was obtained by looking at the reflectivity spectra of the samples before and after compression. The results show a shift in the reflectivity peak after compression, as shown in Figure S3.

#### 3.4.5. Effect of Replacing Water with Ethanol on PCDA Infiltration

**Figure 10:**
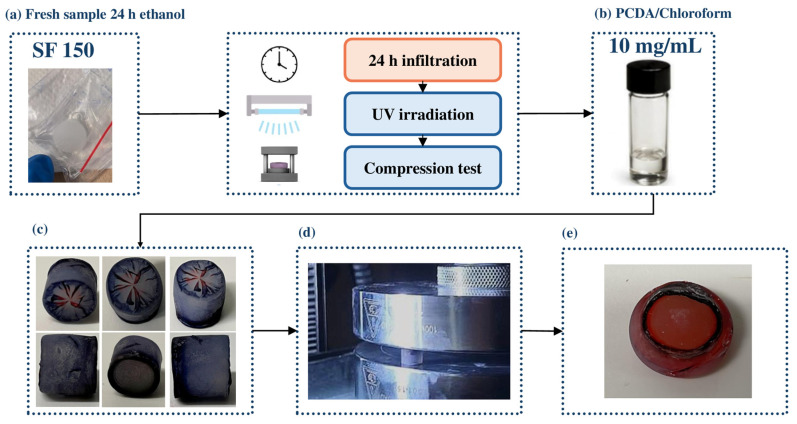
Investigation of the effect of replacing water with ethanol on PCDA infiltration into silk fibroin gels (Protocol P6). (**a**) Fresh SF 150 sample after immersion in ethanol for 24 h to facilitate the replacement of water within the gel network. (**b**) PCDA solution in chloroform (10 mg/mL) used for infiltration. (**c**) Appearance of the sample after 24 h of infiltration in the PCDA/chloroform solution and subsequent UV irradiation, resulting in PDA formation. (**d**) Compression test carried out using an Instron universal testing machine. (**e**) Sample after compression, showing a clear color transition from blue to red, indicative of the activation of the mechanochromic response.

Based on infiltration difficulties observed for fresh samples in previous protocols, we hypothesized that high water content within the gels hindered PCDA impregnation. To verify this hypothesis, a solvent-exchange protocol was developed, in which the gel’s water was partially replaced with ethanol prior to PCDA infiltration (Figure 10).

A fresh SF gel was immersed in ethanol for 24 h to promote solvent exchange within the three-dimensional gel network. The sample was then transferred to a 10 mg/mL PCDA-chloroform solution and allowed to infiltrate for a further 24 h.

Following infiltration, the sample was removed from the solution and subjected to UV irradiation to induce photopolymerization of PCDA. The effectiveness of the infiltration was assessed qualitatively by visually observing the color change and comparing it to that obtained in previous protocols.

Finally, the functionalized sample was subjected to a compression test using an Instron compression cell to verify the mechanochromic response. Improved coloration obtained after ethanol pretreatment provides strong evidence that residual water within the silk network represents a barrier to efficient PCDA deposition. Replacing water with ethanol increases the accessibility of the gel for the chloroform solution, thereby aiding the diffusion of PCDA molecules throughout the matrix. These findings further support the point that solvent exchange, in addition to appropriate polymerization conditions, boosts the effectiveness of the functionalization process.

#### 3.4.6. Discussion of the PDA Functionalization Strategy

The systematic optimization demonstrated that the efficiency of PDA functionalization is primarily governed by the diffusion of PCDA precursor within the silk fibroin network prior to UV-induced polymerization. The results obtained from protocols P1–P5 reveal that both the processing parameters and the physicochemical state of the gel strongly influence the final optical response. Additionally, higher PCDA concentrations resulted in more intense coloration after polymerization, while SF 150 gels consistently exhibited a darker and more homogeneous color. The increase in silk fibroin concentration therefore appeared to promote a more effective retention and distribution of PCDA within the gel network. Furthermore, increasing the infiltration time from 6 h to 24 h and finally to 48 h, as well as reducing the water content within the gel via either drying or solvent exchange, significantly enhanced PCDA uptake, suggesting that residual water limits the ingress of chloroform carrying the precursor. The improved coloration observed after ethanol pretreatment further supports this hypothesis, indicating that replacing water within the gel network facilitates monomer diffusion without requiring complete dehydration. Conversely, prolonged solvent evaporation before UV irradiation did not produce appreciable differences in the final optical response. Based on these findings, SF 150 gels infiltrated with 10 mg/mL PCDA solution for 48 h, followed by brief air drying and UV irradiation, were selected as the optimized functionalization procedure.

Upon mechanical deformation, the PDA network undergoes a conformational transition that produces a distinct color change from blue to red. This mechanochromic response is directly correlated with the applied stress, providing an immediate visual cue.

Preliminary compression tests performed on the optimized PDA-functionalized gels confirmed the occurrence of a visible chromatic transition, demonstrating the successful coupling between the mechanical deformation of the silk matrix and the optical response of the embedded PDA network.

The improved coloration achieved through the optimization process further suggests a more homogeneous distribution of PDA throughout the matrix, which contributes to the consistency and reproducibility of the mechanochromic response.

The intrinsic self-reporting capability of this system makes it attractive for structural health monitoring (SHM) applications. In particular, the possibility of detecting stress concentration, crack initiation, and damage evolution through naked-eye visualization represents a significant advantage for real-time and non-invasive sensing technologies.

It is important to underline that this color shift is irreversible. While it does put certain restrictions on the applicability of the material in SHM, there exist valid scenarios in which having a mechanochromic indicator ‘saving’ the evidence of impact may be beneficial, compared to reversible systems.

## 4. Conclusions

In this work, we demonstrated a simple and effective strategy for the fabrication of silk fibroin (SF) gels directly from Ajisawa reagent through controlled dialysis against an ethanol–water mixture. This approach enables the formation of physically crosslinked gels without the need for chemical modification or complex processing steps. The resulting gels exhibit a homogeneous network architecture governed by β-sheet crystallites, which act as stable physical crosslinking domains, ensuring robust mechanical performance and excellent water stability.

A key finding is the strong dependence of mechanical properties on the initial fibroin concentration, with higher concentrations leading to increased stiffness and compressive strength while maintaining significant deformability. Structural investigations confirmed the prevalence of the silk II conformation, highlighting the effectiveness of the dialysis protocol in promoting controlled β-sheet formation through the progressive removal of Ca^2+^ ions and the synergistic role of ethanol in inducing protein rearrangement.

The system also exhibits remarkable stability in aqueous environments, attributed to the presence of crystalline β-sheet domains, which prevent excessive swelling and dissolution. This feature represents a critical advantage over conventional physically crosslinked hydrogels, particularly for applications in humid or liquid environments.

Importantly, the functionalization of SF gels with polydiacetylene (PDA) introduces a mechanochromic response, enabling the direct visual detection of mechanical stress through a distinct color transition from blue to red. The homogeneous SF matrix ensures efficient stress transfer to the PDA domains, resulting in a reproducible and sensitive optical response.

Overall, the combination of tunable mechanical properties, high water stability, and intrinsic self-reporting capability positions these materials as promising candidates for SHM systems. Although the mechanochromic response observed in this study is irreversible under the investigated conditions, this behavior may still be advantageous for passive damage indication systems, where a permanent visual record of crack formation or overload events is desirable. Future research will investigate strategies to tailor both reversible and irreversible sensing responses depending on the target application.

## Figures and Tables

**Table 1:** Summary of PDA functionalization protocols.

Protocol #	Gel Type	SF Conc. (mg/mL)	PCDA Conc. (mg/mL)	Infiltration Time (h)	Pre-UV Time (h)
P1	Fresh	100, 150	0.7, 10	6	0.1
P2	Fresh	150	10	24	0.1
P3	Dried	50, 150	10	48	0.1
P4	Dried	100, 150	10	48	24
P5	Fresh	150	10	24	0.1

## Data Availability

The original contributions presented in this study are included in the article/Supplementary Material. Further inquiries can be directed to the corresponding author.

## Supplementary Materials

The following supporting information can be downloaded at: https://www.mdpi.com/article/10.3390/polym18151836/s1, Description of the protocol for fabricating silk gels with Ca^2+^ (SF-Ca). Description of the electrical conductivity test on SF-Ca, Figure S1: Photo, XRD, and electrical conductivity of silk gels with Ca^2+^ (SF-Ca); Figure S2: FTIR spectra of silk gels with Ca^2+^; Figure S3: Reflectance spectra of the gels before and after the compression tests.
