# Supplementary material for: Silk Fibroin Gels Fabricated from Ajisawa Reagent with Mechanochromic Sensing Capability
Source: Polymers (Basel). 2026 Jul 27;18(15):1836. doi: 10.3390/polym18151836 (PMC13468121; doi:10.3390/polym18151836)
Supplement: Supplementary file 1 [file polymers-18-01836-s001.zip › polymers-4443716-supplementary.pdf]

Supporting information

To fabricate silk gels with Ca^{2+} (SF-Ca), SF in Ajisawa reagent was dialyzed against water-ethanol mixture once the equilibrium between Ca^{2+} on both sides of the membrane was reached. The gels made from silk solution with Ca^{2+} are referred to as SF-Ca.

Impedance measurements of SF-Ca based gels were performed using Keithley 4200 SCS system. Cylindrical specimens were positioned between two electrical contacts connected via aluminum tape applied to the faces of the cylinder. The electrical resistance values were obtained from the current-voltage characteristics.

The diameter of the gels (D) and height (H) were D = 14 mm, H = 8 mm. All measurements were carried out at room temperature (22 ± 2 °C) and relative humidity of 40 ± 10%. An estimated standard deviation of 2% was observed for all tests.

We further characterized the effect of Ca^{2+} on the secondary structure of SF using XRD analysis (Figure S1a). Once silk was crosslinked, SF gels showed a substantial decrease in beta sheet content. This lower degree of beta-sheet formation may be due to ionic bridge interaction between Ca^{2+} and silk. This phenomenon, in fact, has been shown in previous works [48, 49] and likely prevents the formation of the beta-pleated sheets nanocrystals. While Cl^{-} ions are also present in this system, their effect on protein assembly has previously been indicated to be less relevant [48]. SF-Ca gels exhibited much higher transparency compared to SF gels, evidenced by clear visibility (Figure S1b). This is a typical sign of non-crystalline silk materials too, since the formation of nanocrystals domains is generally accompanied by scattering of the visible light of the resulting gel [45, 50]. In the figure, the dialysis membrane would be positioned at the base of the sample. As gelation progresses in the upward direction, the physically crosslinked bulk of the gel starts affecting the accessibility of the membrane for the remaining SF AJ solution above, and the rate with which the ion gradient equalization is proceeding. This phenomenon is driven by calcium ion concentration remaining relatively high in that segment of the sample.

To further assess the effect of Ca^{2+} ions on the electrical properties of the SF-Ca gels, their conductivity was characterized (Figure S1c). SF-Ca gels exhibited increased electrical conductivity, consistent with the increased availability of water, which enhances the mobility of Ca^{2+} ions.

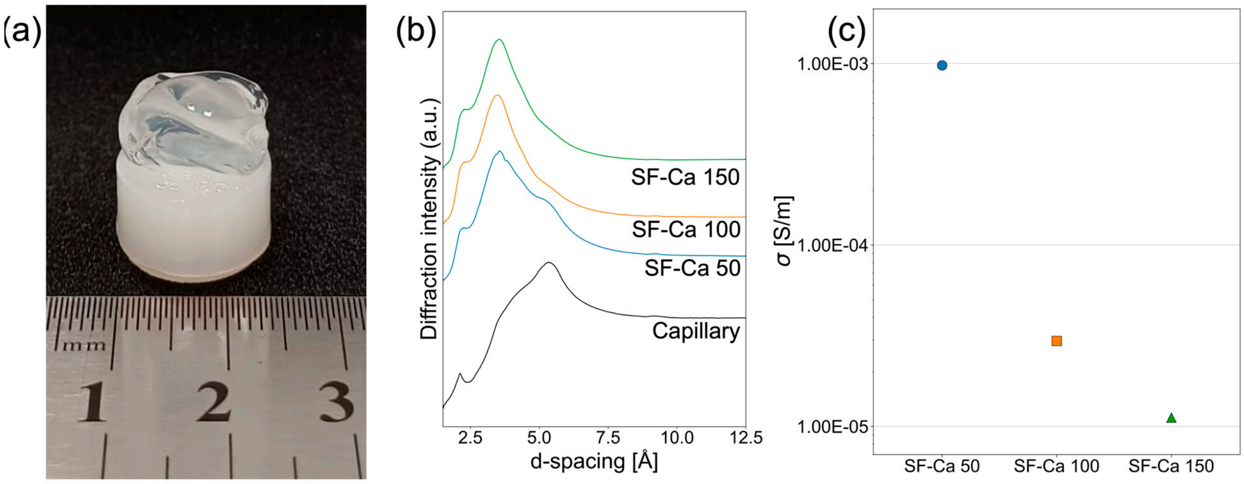

Figure S1. (a) Picture of the transparent SF-Ca gel on top of a SF fresh gel. (b) XRD profiles of SF-Ca gels, showing no peaks associated with Silk II structure. (c) Conductivity measurements of SF-Ca gels.

Dialysis against bath with different $CaCl_2$ concentration was performed in order to characterize to which extent calcium in the bath can impact on the gelation property and secondary structure of the silk fibroin. We observed that at 3% and 6% $CaCl_2$, the solution gelled, while at 10% the gelation didn't occur. FTIR spectra were acquired on the two obtained gels, and a comparison with SF gelled in water was performed. Figure S2 shows optical photogrpahs (a), as well as Amide I and II FTIR spectra (b) and the result of the deconvolution (c). The SF gelled at 3% and 6% amount of $CaCl_2$, but remained liquid af an amount of 10%, as the vial inversion test and optical transparency show, proving that effectively Ca^{2+} ions modulate the gelation of SF. The analysis of the secondary structure showed, moreover, that even though the most abundant conformation remained Silk II, the relative percentage was somewhat lower compared to the ones obtained without calcium.

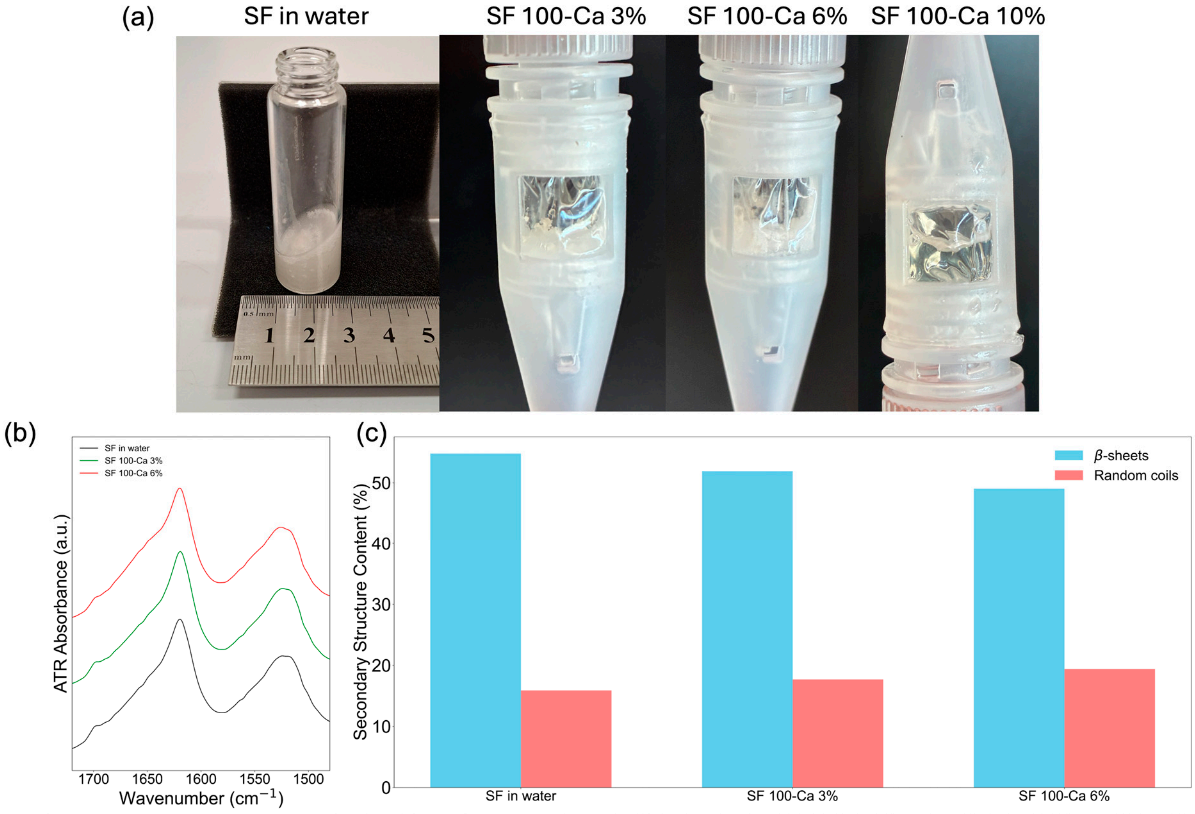

Fig. S2 (a) Photographs of SF gelled from the aqueous solution, and for gels obtained after dialysis against ethanol-water solution with 3% and 6% $CaCl_2$, and after dialysis against ethanol-water with 10% $CaCl_2$ which did not lead to gelation. (b) Amide bands of the FTIR spectra of SF gels in water, 3% and 6% $CaCl_2$. (c) Deconvolution of Amide I bands of panel (b).

In order to prove the shift in color of the gels after the compression test, reflectivity measurements were performed. Reflectance spectra were obtained with an Optosky ATP-910 spectrometer. Before acquiring the

measurements with the samples, black reference and white reference were measured as comparison. The data were collected with the dry gels. The results are shown in Figure S3.

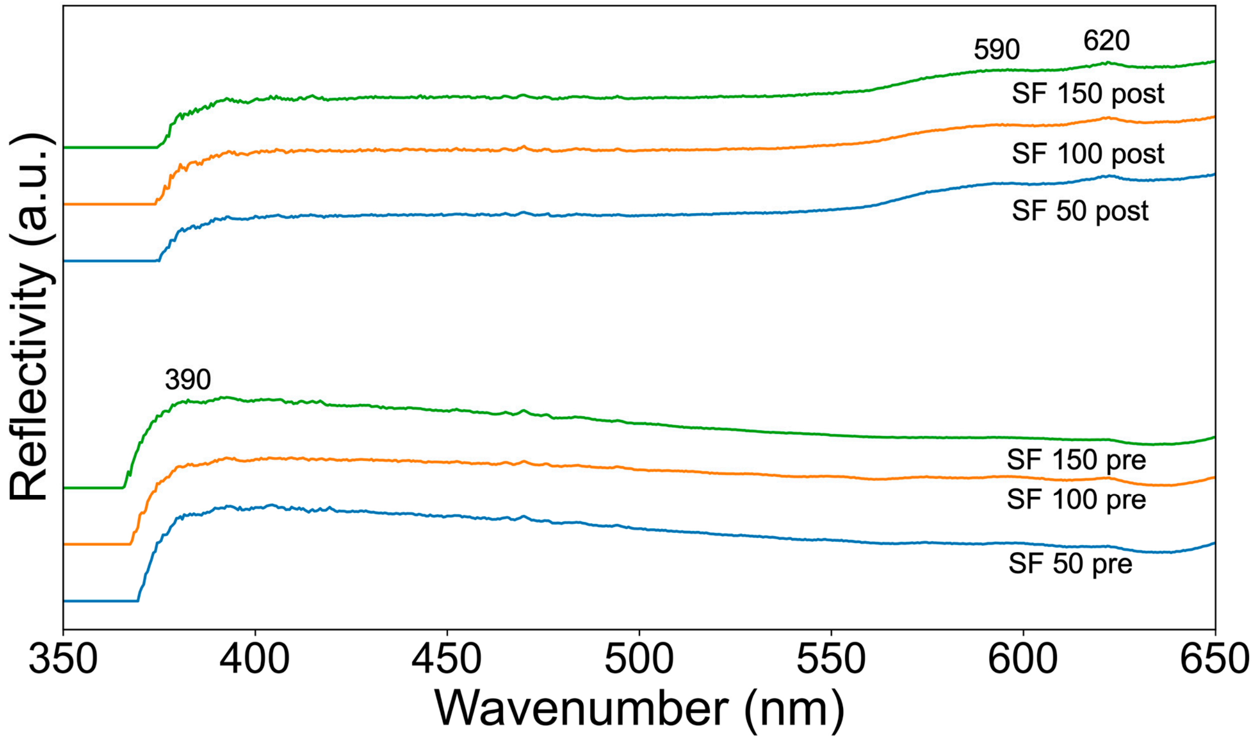

Figure S3. Reflectance spectra of the gels before and after the compression tests, showing the variation of the reflectivity peak. Spectra named "pre" refers to the samples obtained directly after infiltration with PCDA, while the ones named "post" refer to the same samples after compression.
